# Physiotherapists’ delivery of a mobile health physical activity intervention for people post-stroke and transient ischemic attack: Insights from a feasibility study

**DOI:** 10.1177/20552076251374247

**Published:** 2025-09-02

**Authors:** Lucian Bezuidenhout, Sophia Humphries, Coralie English, Carl Johan Sundberg, Michael Nilsson, David Moulaee Conradsson

**Affiliations:** 1 Department of Neurobiology, Care Sciences and Society, Division of Physiotherapy, 27106 Karolinska Institutet, Stockholm, Sweden; 2 Department of Health and Rehabilitation Sciences, Division of Physiotherapy, Stellenbosch University, Cape Town, South Africa; 3 Heart and Stroke Research Program, 5982 Hunter Medical Research Institute, Newcastle, NSW, Australia; 4 Centre of Research Excellence to Accelerate Stroke Trial Innovation and Translation, University of Sydney, Sydney, Australia; 5 School of Health Sciences, University of Newcastle, Newcastle, NSW, Australia; 6 Department of Physiology and Pharmacology, 27106 Karolinska Institutet, Stockholm, Sweden; 7 Department of Learning, Informatics, Management and Ethics, Karolinska Institutet, Stockholm, Sweden; 8 Medical Unit Occupational Therapy & Physiotherapy, Theme Women’s Health and Allied Health Professional, Karolinska University Hospital, Stockholm, Sweden

**Keywords:** Digital health, eHealth, physical exercise, telerehabilitation, secondary stroke prevention

## Abstract

**Background and Purpose:**

Mobile health (mHealth) offers a promising platform for promoting physical activity (PA) in individuals post-stroke or transient ischemic attack (TIA). However, the extent to which key intervention components of PA promotion can be adapted to a digital format remains unclear. This study examines the fidelity of delivering supervised physical exercise and support for individualized PA goals in a 6-month mHealth intervention.

**Methods:**

This feasibility randomized controlled trial included individuals post-stroke or TIA who participated in the mHealth version of the i-REBOUND program. Descriptive statistics were used to assess format (individual/group), and progression of supervised exercise, as well as the characteristics of PA goals (type and use of action planning) throughout the intervention.

**Results:**

Of the 57 participants enrolled in the i-REBOUND program (mean age: 71 years; 70% with stroke), 51 (89%) completed the intervention. Of the 1391 total exercise sessions delivered, the majority (62%) were performed at moderate or higher intensity (Borg rating ≥11). Additionally, 39 participants (71%) demonstrated exercise progression, and 49 participants (89%) participated in group sessions. A total of 293 PA goals were established, predominantly with a focus on performing a desired behavior. Defining frequency (88%), context (61%) and duration (53%) of PA goals was common, while defining intensity (25%) was less so.

**Discussion and conclusions:**

This study support the implementation of a mHealth-delivered supervised exercise program for people post-stroke or TIA, achieving target intensity, integrating group sessions, and progression. Refining goal-setting strategies to address diverse action planning components may enhance future mHealth interventions.

## Introduction

Stroke is one of the leading causes of mortality and disability worldwide,^1^ with mild ischemic strokes being the most common, accounting for approximately 60% of all cases.^2^ The cumulative risk of a recurrent cardiovascular event within 10 years following a stroke or transient ischemic attack (TIA) is 39%.^3^ A TIA causes temporary symptoms without brain damage, whereas an ischemic stroke often results in lasting symptoms and disability.^4^ Low physical activity (PA) is a strong independent predictor of stroke recurrence and regular PA has been shown to reduce post-stroke disability and mortality.^5^ Despite this, low levels of PA are prevalent, even among individuals who have experienced a mild stroke.^6^

Promoting PA requires complex intervention involving various interconnected components,^7^ such as exercise support and behavior change techniques (BCTs). These elements must be carefully adapted to meet the individual's specific needs and the context in which the intervention is delivered. Onsite supervised physical exercise interventions at moderate intensity (e.g. Borg Rating of Perceived Exertion Scale ≥11)^8^ have been shown to improve cardiovascular health.^9^ Supervised exercise, often conducted in a group format to enhance adherence, motivation, and social support,^10,11^ enables close monitoring by the therapist, supporting individualization and progression. Furthermore, PA interventions lasting at least four months and incorporating BCTs have shown promising effects for people post-stroke or TIA.^13^ Effective BCTs for the promotion of PA include structured goal-setting,^13^ including defining intermediate goals^12^ and evaluating and reformulating goals over time.^13^ Goal-setting is further enhanced by incorporating action planning, which defines the where, when, and how to perform the behavior (e.g. context, frequency, duration, and intensity),^14^ aiming to create a concrete plan linked to specific situational cues, thereby promoting consistent behavior execution.

Physiotherapy services for PA often require onsite clinic visits which in turn limits the accessibility and uptake for individuals post-stroke or TIA, especially those in rural areas.^15,16^ Digital intervention, such as mobile health (mHealth, i.e. interventions using tools, like smartphone apps and wearable devices to monitor or treat patients^17^) are a promising solutions for improving access to rehabilitation for people post-stroke or TIA.^18,19^ A 2020 Cochrane review found low-to-moderate evidence that remote services (e.g. mHealth) improve activities of daily living, reduce depression, and enhance quality of life post-stroke, still, few clinical trials have specifically targeted PA.^19^

To evaluate how a complex intervention works, such as multicomponent interventions for PA promotion, it is essential to examine intervention fidelity—whether the intervention core components are delivered as intended in a digital format.^7^ The effectiveness of an intervention may be limited if it is not implemented as intended. Currently, there is a lack of knowledge about the fidelity of evidence-based principles for physical exercise and goal-setting in digital interventions following stroke or TIA. Among the few studies evaluating the fidelity of mHealth post-stroke, findings indicate successful delivery of moderate exercise intensity through video conferencing^20^ and group exercise formats.^21^ However, these studies had small sample sizes and interventions lasting less than 3 months, and none assessed the fidelity of mHealth support for both exercise and goal-setting for PA.

This study builds on the Australian telehealth intervention, i-REBOUND: Let's Get Moving, which supports home-based physical exercise and PA post-stroke.^20^ In our previous work, we have adapted the i-REBOUND program into a fully digital format delivered through a mobile application called STAAR (Stroke Treatment through Active and Accessible Rehabilitation).^22^ Our earlier findings show that the mHealth version of i-REBOUND program was feasible and acceptable, and reached people post-stroke or TIA across Sweden.^23^ With this preplanned secondary analysis, we aimed to evaluate the fidelity of delivering physical exercise and support for individualized PA goals to individuals post-stroke or TIA participating in the mHealth version of the i-REBOUND program in Sweden.

Specific aims were to investigate:

1. Whether the supervised physical exercise was delivered digitally as intended, including transitioning from an individual to a group format, achieving the target intensity of moderate to vigorous PA, and ensuring progression over a 6-month intervention period.
2. The types of PA goals set by the physiotherapist, how they included action planning, and how they monitored participants’ progress in achieving, creating new or refining goals throughout the intervention.

## Methods

### Study setting and ethical considerations

This feasibility randomized controlled trial (ClinicalTrials: NCT05111951) included individuals post-stroke or TIA, who were randomized into either an experimental group receiving the mHealth version of the i-REBOUND program or a control group receiving mHealth-delivered counseling sessions.^24^ This preplanned secondary analysis focused on the delivery of mHealth support for exercise and behavioral change in PA and was therefore limited to the experimental arm of the trial.^24^ Data were collected between September 2021 and May 2023. The study was approved by the Swedish Ethical Review Authority (dnr 2020-05062), and all participants provided written informed consent.

### Participants

Participants post-stroke or TIA were recruited through advertisements in patient organizations, on the Karolinska Institutet website, and via social media. Inclusion criteria were: (a) clinical diagnosis of stroke or TIA three months to ten years before study enrolment, (b) living at home, (c) being able to walk a short distance indoors with or without a walking device, (d) being able to use a smartphone including e-signature identification with/without the help of a relative/carer, and (e) access to a stable internet connection. Exclusion criteria were (a) already meeting the recommended PA levels of at least 150 minutes per week of moderate intensity PA or at least 75 minutes per week of vigorous-intensity PA, (b) severe health conditions compromising engagement in the intervention, or (c) enrolled in a concurrent clinical trial or participating in rehabilitation (e.g. aerobic exercises) at the time of recruitment.

Participant eligibility screening followed a two-step process. First, interested individuals participated in a telephone interview to assess eligibility based on stroke/TIA diagnosis, living conditions, ambulation status, and mobile app proficiency. Second, a video call through the app was conducted to confirm participants’ ability to use the app, follow instructions, and maintain attention. Final inclusion decisions were made based on this screening and a doctor's certificate confirming the diagnosis and no contraindications that would limit participation in an exercise intervention.

### Intervention—mHealth version of the i-REBOUND program

The mHealth version of the i-REBOUND program is a 6-month intervention using digital support for physical exercise and PA through BCTs.^24^ The program was developed through a cross-sector collaboration involving people post-stroke and TIA, physiotherapists, MedTech representatives and researcher.^25^ Two experienced physiotherapists (≥8 years in stroke rehabilitation) delivered the program via a web-based clinic linked to the STAAR mobile app.^22^ Each physiotherapist treated approximately 30 participants over two periods: fall 2021–spring 2022 (*n* = 15) and fall 2022–spring 2023 (*n* = 15).

#### Physical exercises

In the first week of the intervention, participants attended a start-up meeting with a physiotherapist to review their medical history and discuss resources for home-based physical exercise, followed by a practice session to familiarize them with supervised exercise procedures. From week 2 onward, participants were offered supervised exercise sessions via video conferencing: twice weekly during months 1–3 and once weekly during months 4–6.

Each exercise session began with a 5-minute warm-up, followed by 20 minutes of moderate-intensity exercise. The sessions were structured into four blocks of interval training, alternating between high-intensity exercises and lower-intensity exercises for active rest (see Table 1). Functional movements such as sit-to-stand, squats, marching in place, and side stepping were used, requiring no additional equipment. Participants assessed their perceived exertion during each session using the Borg RPE Scale.^8^ The physiotherapist used this feedback to ensure exercises were maintained at the targeted moderate intensity (Borg RPE = 11–14) and adjusted progression across the exercise level if needed.

**Table 1.** Content of supervised physical exercise sessions.

Level		Intervals^a^				
	Description	Duration (seconds)	Exercise examples	Number	Number of blocks per session	Total dose (min)
**1**	- Standing and seated exercises - Focus on movement quality and stability	High: 30; Low: 30	High: Marching in place, side stepping, seated boxing; Low: Weight transfer while standing	5	4	20
**2**	- Standing exercise only - Higher pace and larger movement amplitude compared to Level 1	High: 45; Low: 15	High: Squats, sit-to-stand, lunges, forward and lateral lunges; Low: Weight transfer while standing, marching in place	5	4	20
**3**	- Standing exercise only - Higher pace, larger movement amplitude, and increased complexity (e.g. combining arm and leg movements) compared to Level 2	High: 60; Low: 15	High: Forward and lateral lunges with arm movements, squats; Low: Marching in place, side stepping	4	4	20

See Thurston et al.^24^ for further details.

a The intervals include switching between a “High” demanding exercise and a “Low” demanding exercise (i.e. active rest).

Exercise sessions began as individual sessions, with physiotherapists transitioning participants to small-group sessions (≤4 participants) via video conferencing, based on mobility level. At least two individual sessions were required before becoming eligible for group exercise. The decision to offer group sessions was based on each participant's ability to safely perform the exercises and follow instructions and considered both safety concerns and individual preferences.

#### Behavior change techniques for physical activity

Two individual counseling sessions were offered in the first month of the intervention. The first session addressed motivation, exercise preferences, and barriers to PA. The second session focused on setting individual PA goals using the SMART model (Specific, Measurable, Achievable, Realistic, Timely).^26^ Participants were encouraged to use an activity diary in the STAAR app to self-monitor their PA, with visual feedback on the percentage of activities completed weekly or monthly. Once a month, the physiotherapist met the participant via video conferencing to follow-up on the PA goals, including goal fulfillment, need for revisions, or for establishing new goals. The Patient Goal Priority Questionnaire, sent out before follow-ups, along with activity diary outputs, informed these discussions. This information was also obtained during the last follow-up meeting (month 6).

### Data collection

#### Demographic data

Digital questionnaires were delivered through the STAAR app, collecting demographic data (age, sex, living situation, employment status, educational level, digital health care experience, comorbidities, stroke or TIA diagnosis, and years since onset). The degree of disability post-stroke was assessed using the modified Rankin Scale,^27^ while self-perceived impact and recovery from stroke or TIA were evaluated with the total score of the eight domains of the Stroke Impact Scale and the Visual Analog Scale (VAS; 0 = no recovery, 100 = full recovery) from the same instrument.^28^ Balance confidence was measured using the total score from the Activities-Specific Balance Confidence Scale, ranging from 0 (no confidence) to 100 (complete confidence).^29^ Self-efficacy for exercise was assessed with the Swedish version of the Exercise Self-Efficacy Scale (0 = no confidence to 90 = complete confidence),^30^ and walking ability was evaluated using the Swedish version of the Generic Walk-12 Scale, with scores ranging from 0 (no walking difficulties) to 42 (greater walking difficulties).^31^ Fatigue was measured using the sum score of the Fatigue Severity Scale, where each item is scored on a 7-point Likert Scale.^32^

#### Intervention fidelity measures

Intervention fidelity refers to the extent to which the intervention was delivered as intended. The different domains of fidelity within the mHealth version of the i-REBOUND program are presented in Table 2. Data on intervention fidelity were collected from the physiotherapists’ documentation of therapist-led sessions (e.g. exercise and individual counselling) throughout the intervention period.

**Table 2.** Intervention fidelity and delivery.

Component	Intended delivery
Supervised physical exercise	
Exercise sessions	- Months 1−3: 2 sessions/week//- Months 4–6: 1 session/week
Exercise format	- After ≥2 individual sessions, physiotherapists were instructed to transition to a group exercise format when deemed appropriate and safe.
Exercise intensity	- Moderate intensity, i.e. Borg RPE scale 11–14
Exercise progression	- Physiotherapists were instructed to modify the exercise level when deemed tolerate and safe by the participants, or if the participants perceived the intensity of exercise lower than the targeted level.
Physical activity goals	
Goal-setting	- Physiotherapist were instructed to set individual physical activity goals addressing action planning domains (i.e. context, frequency, duration, intensity),
Monthly goal follow-ups	- Physiotherapists were expected to follow-up on goal progress, adjust, or set new goals

Borg RPE scale: light = 6–10 points, moderate = 11–14 points, and high = 15–20 points^33,34^

For the physical exercise component, the intervention was designed to support individuals post-stroke in progressing from individual to group exercise sessions, engaging in sessions at moderate to vigorous intensity, and gradually advancing through different levels over the intervention period (Table 2). Outcomes included the exercise format, level of exercise and intensity. For the exercise format, data included the number of individual and group sessions delivered, the number of participants eligible for group exercise, and the number of individual sessions completed before participating in group exercise. For exercise progression, the exercise level (see Table 1) was recorded for each participant throughout the intervention period. Progression was defined as moving from exercise Level 1 to 2, 2 to 3, or from 1 to 3, with the higher level being sustained for at least two consecutive intervention weeks. Intensity levels during supervised exercise sessions were categorized following previous research and the clinical application of the Borg RPE Scale, that is, light 6 to 10 points, moderate 11 to 14 points, and high 15 to 20 points.^33,34^

For the PA goals, action planning domains were expected to be addressed (e.g. context, frequency, duration, intensity), and physiotherapists were expected to follow-up on goals and modify them when appropriate (Table 2). Outcomes related to PA goals included the types of goals set and how these goals were refined throughout the intervention period. Firstly, all goals used across the intervention were assessed using the BCT Taxonomy by Michie et al.,^14^ categorizing them as either behavior-based (i.e. focusing on the behavior to be achieved, such as walking daily for 30 minutes) or outcome-based (i.e. focusing on a desired outcome of the behavior, such as being able to walk for 30 minutes). Additionally, we evaluated the extent to which action planning was defined in the goals through specific context (physical or social, i.e. taking a walk in a park with a family member), frequency (e.g. number of times per week), duration (e.g. minutes), or intensity (e.g. moderate PA).^14^ The type of activity (e.g. exercise, or daily activities) specified in the goals was also documented. Secondly, we assessed whether the physiotherapist followed up on the goals and, if applicable, how they were modified. Specifically, goals were categorized as: (i) unchanged, (ii) refined (e.g. by adjusting frequency, duration, or intensity), or (iii) if new a goal was added. The number of refined goals and newly defined goals from the second individual counseling session (month 1) to the final session (month 6) were used for analysis. It is worth noting that there were no preset criteria for the number of PA goals per participant, as goal setting was individualized and tailored to each participant's needs, abilities, and preferences.

### Statistical analysis

Data were analyzed using IBM SPSS Statistics (version 24.0). Descriptive statistics, including frequencies (percentages) and means (standard deviations), were calculated to summarize demographic characteristics, stroke-related variables, balance confidence, walking ability, fatigue and previous experience in digital health.

The intervention fidelity data were exported from the STAAR app as a .csv file and organized by participants’ intervention weeks (1–24). The physical exercise data were analyzed for each week of the intervention phases and summarized descriptively using numbers and percentages. Participants who dropped out of the intervention were included in the analysis up to the week they dropped out. Descriptive statistics were also used to analyze PA goals from the first follow-up (month 1) through the end of the intervention (month 6).

## Results

### Participant characteristics

**Figure 1.**
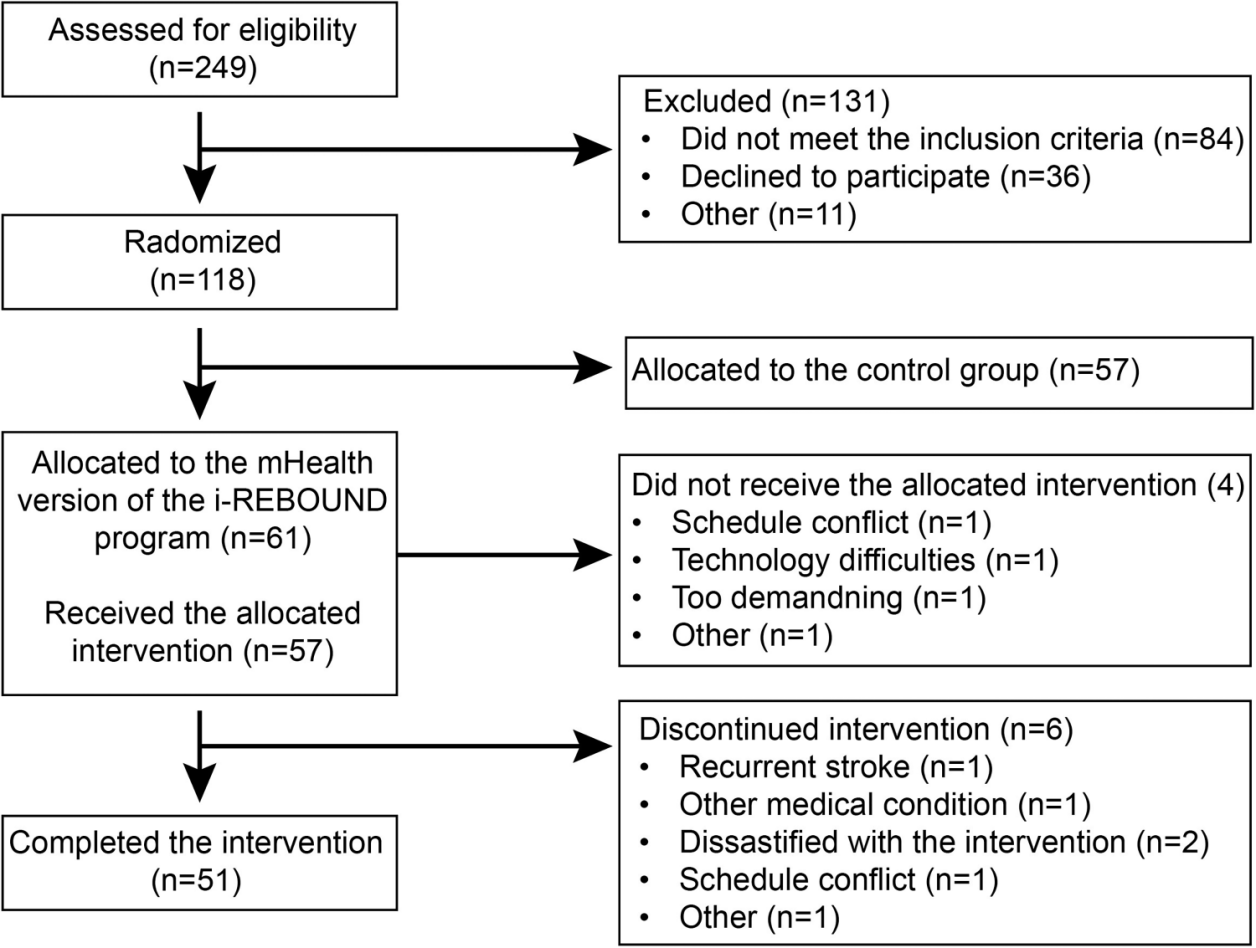
Study flowchart for the mHealth intervention.

At the start of the intervention, 57 people were included in the experimental group with 6 participants dropping out of the program (Figure 1). The majority had a stroke (*n* = 40, 70%) and reported 0–2 on the modified Rankin Scale (*n* = 53, 93%, Table 3). The participants had an average age of 71 years, with the majority being female (*n* = 38, 67%), retired (*n* = 43, 75%), and residing in urban areas (*n* = 40, 70%). A minority used a walking aid (*n* = 18, 32%) and lived alone (*n* = 21, 37%).

**Table 3.** Participant characteristics (*n* = 57).

Age (years), mean (SD)	71.04 (8.5)
Sex male, *n* (%)	19 (33)
Living alone, *n* (%)	21 (37)
Location, urban, *n* (%)	40 (70)
Education, *n* (%)	
Primary school	5 (9)
High school	21 (37)
University degree	31 (54)
Employment status, *n* (%)	
Working	9 (16)
Sick leave	5 (9)
Retired	43 (75)
Stroke, *n* (%)	40 (70)
TIA, *n* (%)	17 (30)
Years since stroke, mean (SD)	3.4 (3.0)
Walking aid (yes), *n* (%)	18 (32)
Modified Ranking Scale, *n* (%)	
No symptoms	10 (18)
No significant disability	43 (75)
Slight disability	3 (5)
Moderate disability	1 (2)
Severe disability	0 (0)
Stroke Impact Scale, mean (SD)	
Total score	36.4 (7.5)
Perceived recovery	70.7 (24.8)
ABC, mean (SD)	74.7 (19.9)
Self-efficacy for exercise, mean (SD)	65.1 (17.6)
Generic Walk-12 Scale, mean (SD)	10.7 (8.2)
Fatigue Severity Scale, mean (SD)	3.8 (1.3)
Previous experience in digital health, *n* (%)	18 (32)

Abbreviations: TIA = transient ischemic attack, ABC = Activities-Specific Balance Confidence.

### Supervised physical exercise

**Figure 2.**
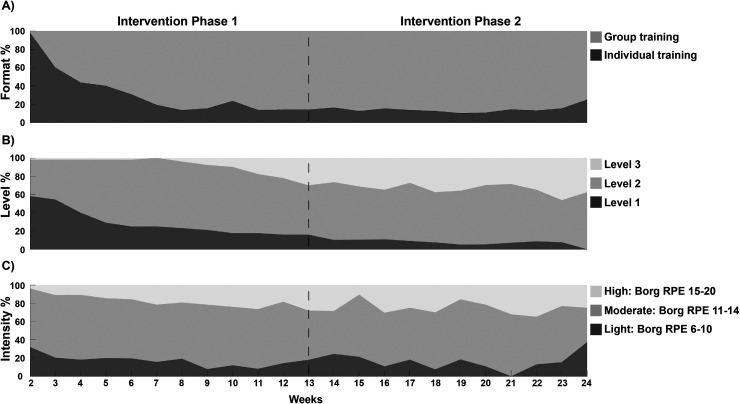
Area plots showing (A) the percentage of participants engaged group and individual training, (B) the percentage of participants at different exercise levels and, (C) the percentage of participants at various exercise intensities throughout intervention. The dashed line indicates the transition between intervention phases 1 and 2.

Figure 2 illustrates the exercise format (Figure 2A), exercise level (Figure 2B) and intensity (Figure 2C) throughout the intervention period. Out of the 1391 total exercise sessions delivered in this study, 69% (*n* = 965) were group sessions, while 31% (*n* = 426) were individual sessions (Figure 2A). Forty-nine participants (89%) engaged in group exercises, whereas 6 participants (11%) only engaged in individual exercises. On average, participants performed 5 individual sessions (min–max: 2–18) before transitioning to group exercises.

Exercise progression was observed throughout the intervention for 39 participants (71%) (Figure 2B). Among participants who made progress, the most common progression across the intervention was from Level 1 to Level 2 (*n* = 22, 56%), followed by Level 2 to Level 3 (*n* = 12, 31%) and Level 1 to Level 3 (*n* = 5, 13%). Among participants who did not make progress, 15 (27%) remained at the same Level (Level 1: *n* = 5, Level 2: *n* = 10), 1 regressed from Level 3 to Level 2, and 3 (5%) fluctuated between levels during the intervention.

The most common weekly exercise sessions during the intervention were performed at moderate intensity (62%), followed by high intensity (21%) and low intensity (17%) (Figure 2C). The exercise intensity was, on an average group level, consistent throughout the intervention.

### Physical activity goals

**Figure 3.**
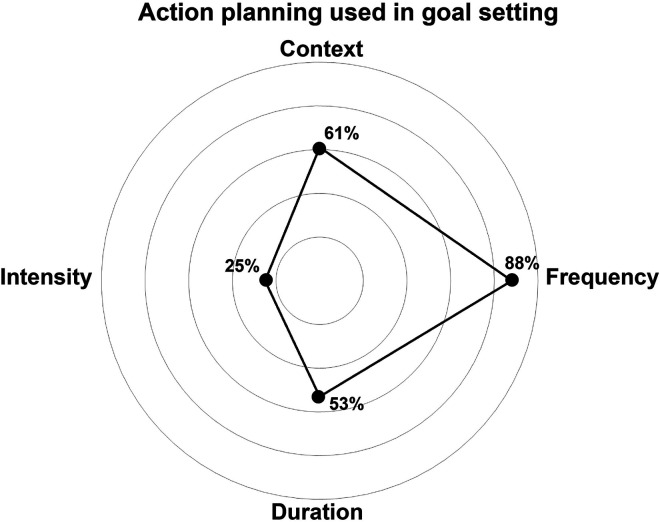
Spider plot illustrating the various domains used for action planning and their respective percentage contributions.

A total of 293 PA goals were set during the intervention, with participants averaging five goals each (range: 1–13). The majority of goals were behavior-related (64%), while outcome goals were less common (18%). A small proportion of goals combined behavioral- and outcome-related elements (15%) or were unclear (2%). The most frequent PA goal types included “other (i.e. housework or swimming)” (*n* = 83, 29%), cardio exercise (*n* = 77, 27%), walking (*n* = 31, 25%), or a combination of cardio and strength exercises (*n* = 37, 12%). As shown in Figure 3, action planning primarily focused on frequency of exercise (*n* = 257, 88%), followed by context (*n* = 178, 61%) and duration (*n* = 154, 53%), with intensity least often specified (*n* = 73, 25%). During the intervention, 46 new goals were created, but refining existing goals was more common, particularly in the early stages (16 refinements in months 1–2) and at the final counseling session (43 refinements at month 6).

## Discussion

This study evaluated the fidelity of delivering the core components of a new mHealth PA program for individuals’ post-stroke or TIA. The findings demonstrated that supervised exercises were implemented as intended, meeting target exercise intensity, incorporating frequent group sessions, and enabling progression in exercise level over time. Most PA goals were behavior-focused (e.g. emphasizing the performance of a specific behavior) and action planning frequently addressed the frequency and context of activities, while duration and intensity were less commonly defined in the goals.

The majority (83%) of completed sessions were conducted at moderate to high intensity, aligning with findings by English et al.,^20^ which used physiotherapist-led sessions via videoconferencing to deliver moderate-intensity physical exercise (median Borg CR10 Score of 3.3) for individual post-stroke. Physiotherapists in our study progressed exercises based on participants’ perceived exertion, movement quality (e.g. postural stability), and safety. Most participants in the present study also successfully progressed across the three exercise levels (71%). Successful exercise adoption regarding intensity and progression was likely supported by participant familiarization with the digital format and the physiotherapists’ knowledge in stroke rehabilitation. Specifically, a familiarization session at the beginning of the intervention was used to ensure safety, technical ease, and adherence to the digital intervention. Although video-based sessions can present technical and communication challenges for people post-stroke, clinical experience is likely of great importance for managing these effectively.^35^

Furthermore, the majority of participants (89%) in the present study participated in group sessions, completing an average of five individual sessions beforehand to facilitate the transition from individual to group exercises. These findings are consistent with Gagnon et al. who reported high levels of adherence and retention of an exercise program, delivered via videoconferencing, that showed promising preliminary results in improving cardiovascular fitness, mobility and strength in community-dwelling stroke survivors.^21^ Group exercise has been shown to enhance adherence and engagement by providing social support, motivation, and accountability, thereby strengthening the establishment of an exercise routine for people post-stroke.^21,36^ In the present study, social interactions between the participants were promoted through verbal communication before and during group sessions. The present findings on mHealth-delivered group exercises, supervised by a physiotherapist, are critical for the future scalability of mHealth trials. Implementing the group format is essential for providing digital support to a large population of individuals post-stroke or TIA in a cost-effective manner.^21,37^

Goal-setting is a recommended BCT for rehabilitation and health promotion post-stroke.^38^ However, barriers to adopting individualized goal-setting persist, including limited patient participation, as well as challenges related to professional skills (e.g. experience in collaborative goal-setting) and priorities (e.g. short- or long-term patient-driven aspirations).^39–41^ Two systematic reviews^39,41^ highlighted a mismatch in goal-setting, with patients often focusing on broad, long-term goals (e.g. regaining function and independence), while healthcare professionals prioritized short-term, impairment-specific goals.^39^ In addition to these challenges, cognitive impairments such as reduced executive functioning, concentration and memory loss can further hinder goal-setting in people post-stroke,^42^ particularly when interventions rely on digital formats that demand sustained attention and engagement. Depression could also limit goal-setting by reducing motivation and engagement in self-management of PA.^43^ Addressing these cognitive and emotional barriers is essential to enhance patient participation and support effective, personalized rehabilitation. In the present study, individualized goal setting was integrated as a key component of the intervention to address individual needs and preferences. Most goals were behavior-oriented (64%), with PA goals varying widely among participants. This variation likely reflects the diverse needs of individuals post-stroke, as they aim to achieve personalized milestones and improvements in PA. Additionally, over time, physiotherapists and participants collectively shifted toward refining existing goals rather than creating new ones. Surprisingly, when developing action plans to meet these goals, duration and intensity were the least specified component, which would suggest information on how long and vigorous an activity was performed was not prioritized.

This study, along with previous feasibility trials,^20,21^ supports the successful adoption of mHealth-delivered supervised physical exercises for promoting PA post-stroke or TIA. The present findings highlight the potential of digital services to enhance PA for people post-stroke or TIA, and ease healthcare burdens,^44^ especially given the high costs of stroke-related comorbidities in Sweden and globally.^30^ However, larger trials are needed to evaluate long-term effects of mHealth on PA and cardiovascular health post-stroke or TIA. If effective, mHealth version of the i-REBOUND program could be delivered at scale and improve access to PA for people post-stroke or TIA, reducing health inequities for those with limited community service access.

## Methodological considerations

The present study reflects the physiotherapists’ perspective on adopting an mHealth intervention for people post-stroke and does not capture the perspective of people living with stroke. Understanding both viewpoints is essential to ensure the intervention is not only feasible from a clinical standpoint but also meaningful and engaging for participants. It is important to consider contextual factors influencing the present results, particularly that the physiotherapists involved in this study had experience in stroke rehabilitation and received training in the mHealth version of the i-REBOUND program prior to the trial. A limitation is that we did not establish predefined criteria for when fidelity would be considered achieved. Future work should establish such benchmarks to enable more rigorous evaluation of intervention delivery. Exercise intensity was primarily measured using the Borg RPE Scale, which reflects participants’ perceived effort. While this is a practical and commonly used approach, it does not ensure that participants were exercising within objective heart rate zones corresponding to low, moderate, or vigorous intensity. However, in contrast to our study which used remote physiotherapy assessments, a recent pilot study using ecological momentary assessment (real-time data collection via smartphone) showed it is feasible to gather patient self-reports of behaviors (where, what activity, and with whom) in people post-stroke.^45^ Our findings compliment those of a prior smartphone study, which demonstrated the feasibility of active behavior tracking related to mood in post-ischemic stroke/TIA patients.^46^ Furthermore, the present study sample included individuals with primarily mild disability post-stroke or TIA, which reflects a sub-group of the stroke or TIA population. For instance, the study sample was about five years younger than the average age for stroke onset of 75 years in Sweden and had a slightly higher proportion of women.^47^ This may limit the generalizability of the findings, as intervention effectiveness and acceptability may differ among male and older stroke survivors. Future research should aim to include more diverse stroke populations to better understand how gender, age and ethnicity may influence impact of this mHealth intervention. There could have also been a risk of selection bias of individuals interested in and aware of PA and health promotion. On the other hand, the present study sample included a diverse group of community-dwelling individuals post-stroke or TIA from various regions in Sweden.

## Conclusion

This study demonstrated the successful delivery of mHealth-delivered supervised exercises, achieving target intensity, integrating group sessions, and supporting progression over time in people post-stroke or TIA. To further enhance PA behavior change, future development should focus on refining strategies for individualized goal setting via mHealth, addressing diverse aspects of action planning, before advancing to a Phase 3 efficacy trial.
